# The Tumor Microenvironment in Follicular Lymphoma: Its Pro-Malignancy Role with Therapeutic Potential

**DOI:** 10.3390/ijms22105352

**Published:** 2021-05-19

**Authors:** Takashi Watanabe

**Affiliations:** Department of Personalized Cancer Immunotherapy, Mie University Graduate School of Medicine, 2-174, Edobashi, Tsu City, Mie 514-8507, Japan; twatanabe@med.mie-u.ac.jp

**Keywords:** follicular lymphoma, microenvironment, follicular helper T cell (Tfh), fibroblastic reticular cell (FRC), follicular dendritic cell (FDC), HVEM/TNFRSF14, T-follicular regulatory cell (TFR), idelalisib, EZH2, TIGIT

## Abstract

In the follicular lymphoma (FL) microenvironment, CXCR5^+^ICOS^+^PD1^+^BCL6^+^ follicular helper T (Tfh) cells, which closely correlate with FL B cells in neoplastic follicles, play a major role in supporting FL. Interleukin-4 secreted by Tfh cells triggers the upregulation of the lymphocyte chemoattractant CXCL12 in stromal cell precursors, in particular by fibroblastic reticular cells (FRCs). In turn, mesenchymal stem cells (MSCs) can be committed to FRC differentiation in the bone marrow and lymph nodes involved by FL. Noteworthy, MSCs can promote the differentiation of Tfh cells into highly immunosuppressive T-follicular regulatory cells. The tumor suppressor HVEM is highly mutated in FL cells, and its deficiency increases Tfh cell frequency. In contrast, PI3Kδ inhibition impedes the recruitment of Tfh/regulatory T cells and impairs the proliferation of follicular dendritic cells (FDCs) and FDC-induced angiogenesis. Since TIGIT ligands are expressed by FDCs, the immune checkpoint receptor TIGIT plays an important role in tumor-infiltrating T cells. Thus, TIGIT blockade might invigorate cytotoxic T cells in the FL microenvironment. Given their potential to simultaneously reduce the neoplastic B cells, Tfh, and TFR cells could also reinforce the effects of the cytotoxic T cells. This combinatory strategy should be explored as a treatment option to tackle FL.

## 1. Introduction

Follicular lymphoma (FL) is the second most common B-cell non-Hodgkin lymphoma (B-NHL), and patients with FL commonly experience a slowly progressive disease. Given its indolent course, approximately 80% of patients already have advanced FL at the initial diagnosis. Patients with FL show a variable clinical path even without treatment, with spontaneous remissions being reported in up to 15% of the patients. Nevertheless, half of FL patients have histologically transformed diseases to aggressive lymphoma and finally succumb to the disease. A variety of treatment strategies for FL are currently used in the clinic, including observation without any treatment (so-called watchful waiting), rituximab alone, the anticluster of differentiation (CD)20 antibody combined with monotherapy or a combination of chemotherapy, radiotherapy in the case of limited disease, radioimmunotherapy (an anti-CD20 antibody conjugated with radioisotope), and high-dose chemotherapy followed by hematopoietic stem cell transplantation. Recently, the cumulative incidence of FL was reduced from 28–45% [1,2] to 9.3% at 10 years as a result of long-term observation following immunochemotherapy with the anti-CD20 antibody rituximab combined with chemotherapy [3]. 

Histopathology of FL is characterized by the formation of neoplastic follicles, which resemble the normal lymph node (LN) architecture. Many non-tumor cells surround the neoplastic cells in the FL LNs, some of which contribute to lymphomagenesis, including LN stromal cells, T-follicular helper cells (Tfh), T-follicular regulatory cells (TFRs), and follicular dendritic cells (FDCs). The correlation between macrophage infiltration in FL LNs and patient prognosis remains controversial, but might vary according to the use of rituximab or different chemotherapeutic regimens [4]. In particular, a doxorubicin-containing regimen has been suggested to abolish the negative effect of CD163^+^ tumor-associated macrophages (TAMs) [4]. As for T cells, intratumoral T cells in FL LNs are heterogeneous depending on the prevalence of various T-cell subpopulations and location of the cells in relation to the follicles. For example, patients with intrafollicular or perifollicular (in a follicular pattern) forkhead box protein 3 (FoxP3)^+^ cells have a significantly higher risk of histologic transformation and shorter survival than those with FoxP3^+^ cells scattered in a diffuse pattern [5]. In contrast, high numbers of tumor-infiltrating FoxP3^+^ cells are related to improved overall survival (OS) in FL [6].

**Figure 1:**
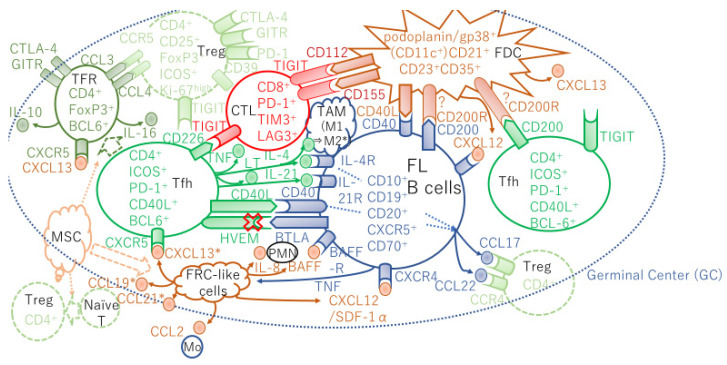
Lymphoid stromal cells and CD4^+^ T cells supporting FL B-cell growth and allowing escape from immune surveillance with cytokine/chemokine circuits in the GC. The disruption of inhibitory signals delivered to the BTLA receptor increases Tfh. HVEM loss trigger production of TNF family cytokines that activate the lymphoid stroma cells; FDCs and FRCs. FRC-like cells produce CCL19 and CXCL13 that recruits CXCR5^+^ Tfh. In turn, Tfh produce IL-4 and IL-21 providing mitogenic signals to FL B cells. TFRs produce CCL3 (MIP-1α) and CCL4 (MIP-1β) are chemotactic for Tregs. * TAM polarization by IL-4 [8] and CXCL13, CCL19, and CCL21 secreted by normal FRCs [9] were results from mice experiments. In mice, TFRs originate from naïve Tregs [10,11,12]. In contrast, human FL TFRs in part originate from Tfh cells [13]. CD200R was found to be expressed just on classical DC (CD11c^+^HLA-DR^+^) from FL LNs. PMN, polymorphonuclear neutrophil; Mo, monocyte. Details in the text.

Programmed cell death-1 (PD-1) is a well-known T-cell exhaustion marker, but two distinct T-cell subpopulations with PD-1 expression were identified in FL. The intratumoral CD4^+^PD-1^high^ T cells, which have a Tfh phenotype, express the C-X-C chemokine receptor type 5 (CXCR5), secrete interleukin (IL)-21, and are B-cell lymphoma 6 (BCL6) positive, but do not express the T-cell immunoglobulin and mucin domain-containing protein 3 (TIM-3), and support B-cell growth. In turn, the CD4^+^PD-1^low^ T cells, which have an exhausted phenotype, express TIM-3 and do not express either BCL6 or CXCR5 (Figure 1) [7]. Furthermore, T cells infiltrated in the FL LN are not simply exhausted, and there are other reasons contributing to their tolerogenic function.

The purpose of this review article is to clarify the peculiar abnormal players in the FL LN compared with their normal counterparts in healthy LNs, with focus on LN stromal cells, especially fibroblastic reticular cells (FRCs), FDCs, Tfh cells, and TFRs. Approaches to invigorate dysfunctional CD8^+^ T cells combined with those to dampen lymphoma-supporting CD4^+^ T cells, if any, and stromal cells are an ideal armament to tackle the incurable FL.

## 2. Heterogeneity of Lymphoid Stromal Cells

FRCs produce and ensheath collagen bundles and other extracellular matrix constituents, organizing an enclosed conduit system that is engaged in the delivery of small molecules, including chemokines and antigens, to the T-cell zone in lymphoid organs. In mice, FRCs express the B-cell-survival factor and T-cell-costimulator B-cell-activating factor belonging to the tumor necrosis factor (TNF) family (BAFF). They produce large amounts of transcripts of the *CC chemokine ligand (CCL) 19* and *CCL21*, and of the *C-X-C motif chemokine (CXCL)13*, a B-cell chemoattractant restricted to FDCs [9]. FRCs transcribe other chemokines, including *CCL2* and *CCL7*, which promote the recruitment or organization of receptor-expressing memory T cells and dendritic cells (DCs). Moreover, FRCs are the main source of the lymphocyte chemoattractant *CXCL12*, also known as stromal cell-derived factor 1 (SDF-1α) [9], and are found in the vicinity of T cells, contributing for their recruitment and dynamic motion along cytoplasmic extensions that function as guide paths. Several studies have reported prominent effects of FRCs on T cell survival [14].

At the earliest stage of germinal center (GC) response, both Tfh cells and a specific FRC subset producing a proliferation-inducing ligand (APRIL) are implicated in the generation of plasmablasts at the GC-T zone interface before terminal maturation into plasma cells [15]. FRCs undergo massive morphological shifts and proliferative expansion to adapt to the increase in B and T lymphocyte numbers, and these alterations trigger the glycoprotein 38/podoplanin (PDPN) on FRCs by C-type lectin-like receptor 2 expression on DCs, leading to reduced FRC contractility [16]. FRCs are also implicated in peripheral immune tolerance, supporting tolerance toward CD4^+^ and CD8^+^ T cells via direct antigen presentation [17] and limiting the proliferation of activated T cells independently of antigen presentation [17]. In mice, this suppressive function was ascribed to the expression of inducible nitric oxide synthase [18], whereas in humans, FRCs compel suppression through a combination of several mechanisms such as indoleamine-2,3-dioxigenase, prostaglandin E2 (PGE2), transforming growth factor-β receptor, and adenosine 2A receptor (A2AR) signaling in secondary lymphoid organs [19]. Indoleamine-2,3-dioxigenase oxidizes tryptophan to kynurenine metabolites, which take away tryptophan from effector T cells, leading to proliferative arrest and exposing them to immunosuppressive kynurenine, thereby impairing T-cell growth and survival [20]. In humans, PGE2 is the most abundant member of the prostanoid family, which is secreted by professional antigen-presenting cells and stromal cells [21]. PGE2 induces a suppressive phenotype in non-regulatory CD4^+^ T cells that can suppress the proliferation of other T cells undergoing activation [22]. Adenosine generation is induced during inflammation and in low-oxygen-tension microenvironments, and A2AR activation elevates intracellular cyclic adenosine monophosphate, which inhibits cytokine responses. Consequently, A2AR repression induces tumor-reactive CD8^+^ T cells in mouse models [23].

Misiak et al. recently showed that FRC-like cells enhance the growth of the entire CXCR5^+^CD4^+^ T-cell compartment, thereby promoting IL-4 production specifically by the PD1^dim^CXCR5^+^CD4^+^ cell subset, in a Notch and intercellular adhesion molecule 1 (ICAM-1)/lymphocyte function-associated antigen-1 (LFA-1)-dependent manner [24]. Analysis of the gene expression profile of FRC-like cells compared with tonsil stromal cells revealed that *ICAM-1* was the most upregulated gene and was highly expressed in FRC-like cell membranes [24]. In mice, ICAM-1 bound to LFA-1 controls Tfh production and maintenance and is involved in the generation of Tfh during helminth infection [25]. ICAM1/LFA-1 pathway in IL-4 overexpression of the cells that correspond to Tfh precursor cells in contact with FRC-like cells [24].

T-cell zone reticular cells (TRCs) are predominant in the paracortex, defining the T-cell zone of LNs and producing the homeostatic chemokines CXCL19 and CCL21 to mediate the recruitment and interaction of C-C chemokine receptor type 7 (CCR7)-expressing naïve T cells and DCs. TRCs keep naïve T cells alive by secreting IL-7 and CCL19 [14].

Marginal reticular cells (MRCs) reside at the edge of the follicle, underneath the subcapsular sinus. A subset of non-FDC CD21-lineage cells are dormant stromal cells that can be converted into CXCL13-secreting cells upon contact with activated B cells of the growing follicles and are called versatile stromal cells [26].

Dark zone CXCL12-expressing reticular cells (CRCs) share with FDCs a high expression of the transcription factor SRX-related HMG-box 9 [27,28]. CRCs are involved in the recruitment of CXCR4^high^ centroblastic B cells in the dark zone, the pole of the GC closest to the T-cell zone.

Medullary FRCs (MedRC) form the major structural components of the plasma cell niche within LN medullary cords [29]. Preadipocytic precursors differentiate into several lymphoid stromal cell subsets, including MRCs and FRCs [30]. Perivascular cells have been proposed as precursors of lymphoid stromal cell subsets, including PDPN/gp38^−^CD31^−^ mural cells (pericytes) expressing smooth muscle actin, platelet-derived growth factor receptor, beta polypeptide, and PDPN/gp38^+^CD34^+^ adventitial cells [31]. Notably, splenic and LN stromal cells appear to rely on different developmental mechanisms and tissue-specific progenitors [32]. MedRCs generate different extracellular matrix structure than TRCs and specifically produce high amount of CXCL12, BAFF, APRIL, and IL-6. They guide the migration of CXCR4^+^ plasma cell within the medulla and contribute to plasma cell survival in situ together with medullary macrophages [29].

## 3. Follicular Dendritic Cell Role in Healthy and Neoplastic Follicles 

FDCs and macrophages are fundamental components of FL pathogenesis [33,34]. Chevalier et al. [35] found that neoplastic follicles included lower DCs and higher regulatory T cell (Treg) frequencies than hyperplastic follicles in control LNs. Treg numbers correlated with the subset of conventional CD11c^+^ DCs, suggesting that the presence of CD11c^+^ DCs in the tumor microenvironment may assist tumor infiltration by Tregs [35]. In case of predominantly diffuse growth pattern of FL, mature PDPN/gp38^high^CCL21^+^ FDCs have been shown to progressively disappear from FL LN, in agreement with the strongly reduced lymphotoxin-α1β2 (LT) production in FL B cells compared with normal GC B cells [36].

In the rituximab era, only few studies have focused on the role of the tumor microenvironment in the transformation of FL. These studies used gene expression profiling or immunohistochemistry to assess the relation between molecular markers in the tumor microenvironment and the time to transformation (TTT). Blaker et al. [37] analyzed paired specimens of FL and transformed FL (tFL) biopsies and compared the cohort with tFL to an independent cohort of FL patients without signs of transformation. Better CD21^+^ FDC meshwork at diagnosis was correlated with shorter OS, progression-free survival (PFS), and TTT in tFL patients treated with rituximab. In addition, the remaining FDC meshworks at transformation were associated with shorter OS and PFS from transformation [37]. 

Moreover, Ohe et al. [38] analyzed the localization of collagen-modifying enzymes on FRCs and FDCs, revealing that the expression of prolyl 4-hydroxylase 1, lysyl hydroxylase 3, and protein disulfide isomerase was often found in FRCs and FDCs in GC in non-neoplastic lymphoid tissues. However, the expression of the collagen-modifying enzymes was lower in most examined lymphomas than in their normal counterparts, and the ratio of transglutaminase (TG) II^+^ FRCs/CD35^+^ FDCs was lower in FL [38]. Interestingly, the same research group found that the proportion of estrogen receptor α on FDCs was higher, along with a larger CD23^+^ FDC meshwork, in Grade 1–2 FL than in Grade 3 FL [39]. Similarly, another group reported that Grade 3 FL exhibited a significant decrease of CD23 expression by the FDCs as compared with G1 FL, although CD21 expression was not significantly different [40]. Furthermore, Ohe et al. reported that high estrogen receptor α expression on FDCs was an independent prognostic factor for OS and PFS in FL patients [41].

Recently, it was shown that FDC-induced genes are relevant to angiogenesis, extracellular matrix formation, and transendothelial migration in a subset of FL samples. The phosphatidylinositol 3-kinase δ isoform inhibitor idelalisib, which was approved for relapsed or refractory (R/R) FL by the U.S. Food and Drug Administration [42], reduced FDC-induced angiogenesis and migration, interference with the FL B-T immunological synapses via CD40/CD40 ligand (CD40L) axis, and affected Treg and Tfh recruitment through CCL22 downregulation [43]. Idelalisib induced the simultaneous reduction of integrins and their ligands in responders, indicating a reduction in the transendothelial migration capability [43]. The CD40/CD40L pathway lies on the crosstalk between B and T cells in the GC and is necessary for B-cell survival, proliferation, and differentiation into plasma cells. Idelasilib inhibits the proliferation of FDCs that overexpress CD40L on their cell membranes, and it decreases the expression of ICAM-1 or CD80, all of which are directly involved in B-T immunological synapses. Moreover, idelalisib decreases the expression of CCL22 secreted in the FL-FDC niche, which is the ligand for the CCR4 receptor expressed on Tregs [43].

Although the B-cell lymphoma 2 (BCL2) inhibitor venetoclax showed limited activity in FL [44], a recovery of venetoclax activity was observed in coculture with FL-FDCs or FL-macrophages when treated with idelalisib, by restoring BCL2 dependence over B-cell lymphoma-extra large (BCL-X_L_) and myeloid cell leukemia-1 or BCL2-related protein A1, respectively [43]. Collectively, combination therapy of anti-B-cell targeting agents with idelalisib, a FL microenvironment modulator should be explored.

Interestingly, digital image analysis of the distribution of bystander cells within follicles of FL revealed that, whereas B cells and macrophages display complete spatial randomness, all T cells, Tfh (identified by PD-1), and DCs (identified by CD21) clustered within a radius of 6–10 μm [45].

A couple of studies providing disruption strategies of FDCs-FL cell crosstalk were reported in vitro. The previous study indicated that FL cells having cancer stem cell-like activities interacted with FDCs in a CXCL12/CXCR4 dependent manner, including resistance to cyclophosphamide or doxorubicin, in vitro and in NOD/Scid mice and that a specific CXCL12/CXCR4 inhibitor exhibited tumor growth [46]. Furthermore, PI3K inhibitors can be one of the candidate drugs combined with antineoplastic B cell agents because a pan-PI3K inhibitor showed counteractions against angiogenesis, cell adhesion, migration, and “serum-like responses” (namely, cell survival and proliferation [47]) activated by FDCs [48]. However, there is no information on the selective PI3Kδ isoform inhibitor idelalisib against these activities. There have been no reports concerning targeting FDCs-FL interaction since 2015 either.

## 4. Mesenchymal Stem Cells Orchestrate the FL Cell Niche and Cancer-Associated Fibroblasts in the FL Microenvironment 

Cancer-associated fibroblasts (CAFs) are phenotypically and functionally different from their normal counterparts, presenting a niche-based model of oncogenesis attributed to the dynamic coevolution of both cancer and stromal cells. Mesenchymal stem cells (MSCs) can be recruited within tumors, where they are integrated into the stroma, become activated, and augment tumor growth. LN containing bona fide MSCs, human bone marrow (BM)-MSCs, and LN-MSCs can be committed to FRC differentiation in response to a combination of TNF-α and LT, the two main factors implicated in the differentiation and sustenance of secondary lymphoid organs. Mesenchymal cells recruit malignant B cells and protect them from spontaneous and drug-induced cell death [49,50]. LT and TNF-α are two non-redundant key factors involved in lymphoma stromal cell differentiation and maintenance. B cells contribute to FRC activation and maintenance in both LN and spleen through their inducible expression of LT [32,51,52].

MSCs was shown to induce the differentiation of naïve T-cells to Tregs, thereby contributing for the modulation of the FL biology [53]. Thus, MSCs were proposed as organizers of the FL cell niche. MSCs overexpress CCL2, which recruit monocytes. Monocytes are further changed into proangiogenic and anti-inflammatory macrophages [54]. In particular, FL B cells can trigger the commitment of MSCs to differentiation into an FRC-like phenotype, and for MSCs to overexpress CCL2 and IL-8 in a TNF-dependent manner, resulting in FRC meshwork activation within involved LN and BM [49,54,55]. Furthermore, overexpression of IL-4 induces a TG ^high^PDPN/gp38^low^gp38lowvascular cell adhesion molecule-1 (VCAM-1)^high^CXCL12^high^ phenotype in human mesenchymal progenitors and FRC-like cells, a profile that resembles that identified in situ within the FL cell niche [56]. Additionally, TNF-α/LT and IL-4 induced the construction of an extracellular TG-positive meshwork. Whereas TNF-α/LT decreased TG mRNA, IL-4 induced both TG expression and redistribution at the surface of stromal cells [56]. Additionally, TNF/LT and IL-4 can induce the construction of an extracellular TG^+^ meshwork [56].

The upregulation of CXCL12 from FL-CAFs in FL-infiltrating LN and BM contributes to the migration, adhesion, and activation of FL B cells with a CXCR4^+^ phenotype [56] (Figure 1). BM obtained from FL patients with BM involvement revealed CD20^+^ FL B-cell aggregates with paratrabecular localization, which is characteristic of BM involvement of FL. These B-cell-infiltrated regions exhibited elevated CXCL12 expression compared with outside regions of FL infiltration [6]. FL B cells secrete TNF-α, previously involved in the induction of both CCL2 and IL-8 production by BM-MSCs [54,55]. Stromal cells cocultured with TNF^high^ malignant B cells induced a decrease in CXCL12 expression. Conversely, purified CD4^+^CXCR5^high^PD-1^high^ FL-Tfh cells induced elevation of CXCL12 in adipose tissue-derived stroma cells [56].

The specific downregulation of LT in FL B cells combined with upregulation of CXCL12 in FL-CAFs suggest a CRC-like origin of at least some FL stromal cells since dark zone CRCs, unlike FRCs and versatile cells, do not require LT and TNF-α to maintain CXCL12 expression and the network morphology [27]. Cultured FL BM stromal cells retained numerous features of their native counterparts, including the overexpression of CCL2, IL-8, and CXCL12, suggesting an imprinting of the stromal cells by the tumor context [54,55,56].

Autologous CAFs or the stromal cell line HS-5 were found to protect primary FL cells from apoptosis in response to the BCL2 inhibitor ABT-737 through mRNA induction of the adenosine triphosphate-binding cassette (ABC)-drug transporter genes *ABC subfamily C member 1 (ABCC1)* and *ABC sub-family G member 2 (ABCG2)*, and upregulation of *BCL-X_L_* [57]. Furthermore, Sakamoto et al. demonstrated that pyruvate secreted from patient-derived CAFs supported the survival of primary FL cells [58].

## 5. Follicular Helper T Cells

Unlike other helper T-cell compartments, follicular CD4^+^ T cells have been defined by their localization in secondary lymphoid organs at the T/B border or within B-cell follicles. Human Tfh cells are defined by the expression of the chemokine receptor CXCR5, the inducible T-cell co-stimulator (ICOS), and PD-1. Tfh cells express the transcription repressor BCL6 (Figure 1) (Table 1), and support the survival and differentiation of normal GC B cells [59]. The Tfh cell compartment comprises a greater proportion of CD3^+^ T cells in the FL LNs than in normal LNs [60]. Ame-Thomas et al. demonstrated that Tfh cells support FL B-cell viability through the secretion of IL-4 and the expression of CD40L [60]. Moreover, intratumoral Tfh cells expressing IL-4 and CD40L can also induce the production of CCL17 and CCL22 by FL cells (Figure 1), thereby promoting the active migration of Tregs (Figure 1) and IL-4 producing CD4^+^ T cells. Furthermore, IL-4 considerably enhanced phosphorylation of signal transducer and activator of transcription 6 (p-STAT6) in primary FL cells [61]. In fact, Pangault et al. demonstrated that the majority of p-STAT6^+^ B cells were localized in close proximity to the cells that expressed PD-1^+^ (Tfh) [62]. Additionally, it was recently demonstrated that PD-1^+^ICOS^+^ Tfh cells and proliferating Ki-67^+^ tumor cells are in close contact, and that immune synapses are formed in FL [63]. Moreover, immunohistochemistry studies of FL LNs revealed purified CD4^+^CXCR5^high^PD-1^high^ Tfh cells, which produced IL-4 and triggered an upregulation of *CXCL12* in adipose-derived stromal cells. This IL-4/CXCL12 loop can be magnified in activated lymphoid stromal cells, as displayed in an in vitro model of human lymphoid stromal differentiation and in an inducible mouse model of ectopic lymphoid organ formation [56]. Furthermore, CXCL12 induced primary FL B-cell migration, adhesion to stroma cells, and activation demonstrated phosphorylation of the spleen tyrosine kinase (Syk), another key mediator of B-cell antigen receptor signaling, and its downstream target extracellular signal-regulated kinase (ERK) [56]. FL B-cell migration was similarly reduced in response to a CXCL12 receptor CXCR4 (Figure 1) inhibitor, the Bruton’s tyrosine kinase (BTK) inhibitor ibrutinib, and idelalisib [56]. 

In mice, Luthje et al. showed that Tfh cells were not terminally differentiated and retained the flexibility to be mobilized into other helper T cell subsets [64]. They showed that Tfh cells are multifunctional helper T cells that produce interferon (IFN)-γ, IL-2, and IL-4. These cells proliferate and produce transferrable memory cells with plasticity, which in turn differentiate into conventional effector helper T and Tfh cells after recall following viral infection [64]. Furthermore, Ame-Thomas et al. identified that the CD10^+^ subset of Tfh cells from the FL LNs have an IFN-γ^low^TNF-α^high^ cytokine profile, which favorably induces and maintains a B-cell supportive lymphoid stromal network linked to a strong capability of support autologous neoplastic B-cell survival in vitro [65]. CD10 is a marker of immature T and B cells. Although Tfh cells obtained from FL secrete high levels of IL-21 and low levels of IL-17 compared with Tfh cells from the tonsils. The CD10^+^ subset of Tfh cells produces high levels of IL-4 that can cause B-cell activation, survival, and production of CCL17 and CCL22, which recruit Treg (Figure 1) [65]. IL-4 also contributes to TAM polarization (Figure 1) [8]. Thus, creating a vicious cycle, Tfh cells seem to play a role in constructing an immunosuppressive FL microenvironment that accelerates immune escape and FL growth and survival. Blaker et al. showed that high intrafollicular CD4^+^ T cell scores at diagnosis were associated with shorter TTT [37].

Interestingly, T-cell receptor repertoire analysis in a Tfh cell subset revealed that the clonality of T cells in follicular areas was greater than that in the interfollicular area in FL LN, with the more frequent clones predominating in the follicular regions as compared with the interfollicular areas [63].

Recently, single-cell transcriptomic data demonstrated that *cathepsin S (CTSS)* is significantly overexpressed in FL cells as compared to centroblasts and centrocytes from normal GC [66]. Approximately 40% of FL patients exhibited intermediate-to-high levels of CTSS protein in 10–80% of FL cells. CTSS can be activated by recurrent Y132D mutations and overexpressed in FL, suggesting a specific and selective role of CTSS in FL lymphomagenesis. In the vavP-Bcl2 chimeric model, histopathological analyses of *CTSS^high^* and *CTSS^Y132D^* tumors showed characteristic features of FL and a significant increase in CD4^+^ Tfh cells. In the animals that overexpressed CTSS or the mutant CTSS, CD8^+^ T cells were prone to be eliminated and CD4^+^ T cells were highly infiltrated and colocalized with tumor B cells. The oncogenic activity of CTSS depends on its capability to accelerate crosstalk between FL cells and Tfh cells; thus, depletion of CD4^+^ T cells by an anti-CD4 antibody abolishes the ability of vavP-Bcl2-*CTSS^high^* and *CTSS^Y132D^* to develop tumors due to impaired GC B cell differentiation [66].

Chimeric antigen receptor (CAR)-T cells have demonstrated to be effective against R/R FL [67,68], tFL [69,70], and diffuse large B-cell lymphoma [68,69,70]. To further target Tfh cells and B-cell lymphoma cells, CAR-T cells targeting CXCR5 have been recently designed with demonstrated comparable activity with conventional CD19 CAR-T cells [71]. However, CXCR5^+^CD8^+^ T cells are present in human tonsils and FL and exhibited strong cytotoxic activity, although the mean fluorescent intensity of CXCR5 in CD8^+^ T cells was much lower than B cells and CD4^+^ T cells in human tonsils and FL specimens [72]. Therefore, there is a possibility that the CXCR5 CAR-T might cause fractricide of efficient cytotoxic T cells. This may explain CXCR5 CAR show similar antitumor activity to CD19 CAR, although they used mantle cell lymphoma cells not FL cells implanted in NOD/Scid/IL2Rγnull mice [71].

On the contrary, since PI3K is essential for the generation and function of Tfh from the results that p110δ subunit is pivotal for ICOS downstream signaling and the production of key Tfh cytokines; namely, IL-4 and IL-21 [73]. Therefore, idelalisib can be one of the potent molecular target drugs for CD19 CAR-T cells efficiently to operate, dampening the protumoral components in the FL microenvironment.

## 6. The Herpes Virus Entry Mediator/B- and T-Lymphocyte Attenuator Axis

*Tumor necrosis factor receptor superfamily 14 (TNFRSF14)*, which encodes the herpes virus entry mediator (HVEM), is the fourth most highly mutated gene in FL [78]. HVEM limits T-cell activation via the ligation of the B- and T-lymphocyte attenuator (BTLA). BTLA contains an immunoreceptor tyrosine-based inhibition motif (ITIM) in its cytoplasmic domain that can recruit the Src homology domain 2 (SH2)-containing protein tyrosine phosphatase-1 (SHP1) and SHP2 [79]. BTLA-HVEM at the immunological synapse recruits SHP1 to inhibit signaling downstream of the T cell receptor in Tfh cells [80]. *HVEM* localizes to the minimal common region of the chromosome 1p36 deletion, which is associated with worse prognosis in FL [81]. In a vavP-BCL2 FL mouse model, in which hematopoietic progenitor cells isolated from fetal livers were transduced with retrovirus expressing short hairpin RNAs to knock-down *Hvem*, tumors demonstrated significantly increased expression of FDC- and FRC-derived cytokines; namely, CXCL13 and CXCL19, compared with control tumors transfected with vector alone [78]. Since the stroma-derived cytokine CXCL13 is the main chemoattractant for CXCR5^+^ Tfh cells (Figure 1) [82], a significant increase in the proportion of Tfh cells secreting a high quantity of IL-4, TNF-α, and LT (Figure 1) in HVEM-deficient tumors was observed compared with control tumors [78]. Moreover, immunohistofluorescence staining demonstrated a significant increase in the CD21^+^CD35^+^ FDC network (Figure 1) within follicles in HVEM-deficient tumors compared with control tumors. Similarly, as a result of the activation of FRCs, the intensity of type I collagen in perifollicular regions was significantly elevated in HVEM-deficient lymphomas [78]. Human GC-derived Tfh cells are characterized by high expression of the BTLA receptor [78]. The Tfh-derived cytokine IL-4 phosphorylates STAT6 and significantly increased STAT6 phosphorylation was found in HVEM-low human FLs [78]. Thus, in HVEM-deficient FLs, Tfh cell recruitment and B cell activation were observed.

In particular, *HVEM/TNFRSF14* loss was found in 30–40% of FL patients [78,83]. In addition, HVEM^−^ FL patients exhibited greater expansion of Tfh cells than HVEM^+^ FL patients. Using mouse B-cell lymphoma cells, a soluble HVEM ectodomain protein fragment blocked BTK phosphorylation, similar to ibrutinib. Furthermore, soluble HVEM inhibited the B-cell receptor signals via Syk and the B cell linker, and ERK phosphorylation in primary human FL B cells and in DoHH2 human lymphoma cells derived from tFL that expressed BTLA and carried a homozygous *HVEM* deletion. Moreover, since it prevents the growth of aggressive Myc^+^Bcl2^+^ murine lymphoma cells that express BTLA and lack HVEM in vivo, CAR-T cells that restore loss of *HVEM* are a promising therapeutic strategy [78].

In contrast, mutations or deletions in *BTLA* were not found [78]. Since *BTLA* expression is controlled by the histone lysin N-methyltransferase 2D (KMT2D), it is plausible that reduced *BTLA* expression in FL may be attributed to KMT2D inactivation [78], as truncating and missense mutations affecting *KMT2D* were observed in 60–70% of FL patients, and loss of *KMT2D* results in decreased global lysine 4 on histone H3 methylation levels and downregulation of key genes involved in immune signaling and B cell differentiation [84]. However, BTLA expression in the intrafollicular area was found to be a marker of Tfh cells, whereas its expression in the interfollicular area was a marker of T cells in FL, with high BTLA expression being correlated with a favorable overall survival in FL [85]. In addition, tFL was characterized by a remarkable decrease in the number of BTLA^+^ cells. Furthermore, high TNFRSF14 expression was found to be correlated with poor OS and PFS. In addition, TNFRSF14^high^ correlated with B-symptoms, high β2-microglobulin, and high-risk groups according to the FL international prognostic index [85]. Since the interaction between the HVEM and BTLA receptors is ablated in the majority of FLs, modified CD19-targeted CAR-T cells that enable locally compensate the soluble HVEM were significantly more effective than conventional CD19-targeted CAR-T cells against lymphoma cells [78].

Recently, interesting findings have been reported concerning epcoritamab, a novel bispecific IgG1 antibody redirecting CD3^+^ T cells to CD20^+^-expressing tumor cells. An ongoing first-in-human clinical trial on epcoritamab showed a favorable safety profile and preliminary efficacy data indicating encouraging antitumor activity as a single agent in patients with R/R B-NHLs, including FL [86]. Noteworthy, van der Horst et al. [87] found no association between T-cell activation and tumor expression of CD20, PD-L1, or HLA-DR. Activation of allogenic CD4^+^ and CD8^+^ T cells was the highest in patients that showed low expression of HVEM in B-NHL cells, including FL. T-cell activation was significantly lower in patients with higher tumor HVEM expression. However, no relationship was observed between HVEM expression and epcoritamab-dependent cytotoxicity [87]. This corroborates the previous findings that frequencies of polyfunctional alloreactive T-cell, which was defined combining intracellular measurements of IFN-γ, IL-2, or TNF-α and surface expression of CD107a, after stimulation with FL B cells harboring dual aberrations (for example, homozygous deletions, nonsense mutations and deletions) were higher than FL B cells with wild-type *TNFRSF14* [83]. This implies that HVEM^−^ FL B-cells seem to be immunogenic.

## 7. T-follicular Regulatory Cells

Tregs expressing T-bet can upregulate CXCR3, urging them to traffic to sites of Th1 inflammation and thus suppress Th1 cells. FL-infiltrating Tregs have been demonstrated to potentially inhibit proliferation and the cytokine production of FL-infiltrating T cells [74]. Tregs that localize in the GC are a distinct subset of Tregs called TFRs [10,11,12]. T-cell population is characterized by the dual expression of FoxP3 and BCL6 (Table 1) [10,11,12,60,75]. In mice, TFRs originate from naïve Tregs that upregulate BCL6 upon activation, leading to CXCR5 expression that direct TFRs to the GC through gradients of CXCL13 [10,11,12]. In contrast, human FL TFRs in part originate from Tfh cells [13], supporting FL B-cell viability [60]. MSCs promote the differentiation of Tfh cells into TFR by inducing the expression of FoxP3 [13]. MSCs support the viability of FL-infiltrating Tfh cells in part through an IL-6-dependent mechanism [13]. TFRs express a cytokine/chemokine profile that is unique compared with Tregs in normal LN. Cell migration and movement categories were highly enriched in FL LNs compared with normal LNs. Significant differences were found in cytokine, chemokine, G-protein receptor, and adhesion molecule genes. Moreover, RNA sequencing data revealed that TFR has greater suppressive capacity than Tregs in normal LN or reactive LN by upregulating *cytotoxic T-lymphocyte-associated protein 4 (CTLA-4)*, *IL-10*, and *glucocorticoid-induced tumor necrosis factor-related protein (GITR)*, all of which were confirmed by protein expression [76]. IL-16 is also abundantly secreted by TFRs compared with normal LN, although IL-16 preferentially attracts Th1 and Treg subsets. In addition, they showed that TFRs produce more C-C motif chemokine ligand 3 (CCL3) (also known as macrophage inflammatory protein-1 (MIP-1)α) and 4 (CCL4) (also known as MIP-1β)), chemoattractants for CCR5-expressing Tregs, and CD4^+^ and CD8^+^ T cells, than Tregs in normal LNs. Furthermore, TFRs express *CXCL13,* which suggests that there is an autocrine feedback that resulted in TFR retention in the GC. TFRs localize and accumulate within malignant LNs through the downregulation of *SELL* (encoding L-selectin), *CCR7*, and *sphingosine-1-phosphate receptor 1*, a type 1 G-protein coupled receptor that is a primary determinant of lymphocyte egress from the LN. The *SELL* expression in TFRs was lower than that in normal LNs, suggesting TFR retention in the GC [76].

Tregs usually express the IL-2 receptor α chain CD25, but lack the IL-7 receptor α chain CD127. Le et al. showed that CD25^high^CD127^low/−^Tregs were accumulated in FL samples, and that the proportion of ICOS^+^ Tregs among CD4^+^ T cells was significantly increased in FL tumors [77]. Compared with ICOS^−^ Tregs, FL tissues showed significant augmentation of surface CTLA-4, GITR, CD39, PD-1, and higher Ki67, which suggested that activated Tregs expanded in the FL microenvironment [77]. A decrease in ICOS ligand (ICOSL) expression in FL B cells was observed in the presence of ICOS^+^CD4^+^ T cells in vitro, and antagonist anti-ICOS antibody restored the high expression of ICOSL in FL B cells, suggesting that the ICOS/ICOSL interplay is involved in the downregulation of ICOS expression in FL B cells [77]. ICOS expression was markedly higher in CD25^high^FoxP3^high^ Tregs, which were shown to produce less IL-2 and IFN-γ than CD25^+^Foxp3^+^ Tregs. Therefore, when a neutralizing anti-ICOS or anti-ICOSL monoclonal antibody was added to cocultures of CD4^+^ T cells and FL B cells, a significant reduction in the percentage of CD25^high^FoxP3^high^ Tregs was observed [77]. Since about 25% of conventional T cells in mononuclear cell extracts from FL samples are Tfh cells characterized by ICOS and CXCR5 expression [77], the ICOS/ICOSL pathway between FL-supporting Tfh and FL B cells can be sensitive to treatment with anti-ICOS/ICOSL antibody as well.

One of the potential approaches to deplete Tregs is the anti-CTLA-4 antibody. The results of a phase I study on ipilimumab combined with rituximab in R/R B-NHL patients, where FL was enrolled as the highest population (39%), showed manageable toxicities and encouraging efficacy. In this trial, the ratio of CD45RA^−^Tregs to total Tregs was significantly increased in responders compared to non-responders over the examined period of time (days 0, 8, 15, and 70) [88], although Tregs in peripheral blood do not reflect Tregs in LN, especially TFRs.

Furthermore, epigenetic regulator zeste hololog 2 (EZH2) is a catalytic subunit of polycomb repressive complex 2 that trimethylates lysine 27 on histone H3 [89], resulting in gene repression, and EZH2 can form a complex with FoxP3 that is fundamental to maintaining the identity of naturally occurring Tregs following its activation [90]. The modulation of the EZH2 expression in T cells can improve antitumor responses elicited especially in the presence of the anti-CTLA-4 therapy. Blockade of CTLA-4 signaling enhanced *EZH2* expression in human CD4^+^ effector T cells and CD8^+^ T cells and Tregs, whereas EZH2 inhibition improves response to anti-CTLA-4 via modulation of cytotoxic effector T cells and altering the Treg phenotype into effector-like T cell profile [91]. Recently, tazemetostat, a first-in-class, selective, oral EZH2 inhibitor, which inhibits either wild type or mutant, showed clinically durable responses in heavily pretreated patients with R/R FL [92], although activating mutations of *EZH2* are present in 20–30% of patients with FL and tFL [93,94]. Intriguingly, EZH2 expression is elevated in tumor-infiltrating Tregs, and pharmacological inhibition of EZH2 destabilize FOXP3 expression and slows tumor growth through enhancement of the recruitment and function of CD8^+^ and CD4^+^ T cells [95]. Thus, tazemetostat target FL B-cells with recurrent *EZH2* gain-of-function mutations and modulate FOXP3 expression in TFRs. Furthermore, idelalisib was recently shown preferentially to inhibit human Tregs compared with CD4^+^ and CD8^+^ effector T cells [96]. Collectively, these kinds of the functional TFR modulators combined with anti-B-cell drugs should be explored.

## 8. Other T Cell Dysfunctions in FL

The immunomodulatory drug lenalidomide (Revlimid) combined with rituximab (R^2^ immunotherapy) was recently approved for relapsed or refractory indolent B-cell lymphoma, including FL [97]. By the combination of lenalidomide with rituximab, restoration of appropriate F-actin immune synapse formation was confirmed by confocal images of clinical samples obtained from LN biopsies conjugated with circulating tumor B cells obtained from patients with leukemic state of FL, accompanied with polarized expression of granzyme B at CD8^+^ T cell contact sites and synapses with FL B cell and with tyrosine phosphorylated proteins in CD4^+^ T cells [98].

Recently, T-cell immunoglobulin and ITIM domain (TIGIT) has been highlighted as a coinhibitory receptor in FL. The majority of CD8^+^ effector memory T cells from lymphoma coexpress TIGIT and PD-1 as compared to tonsillar cells (Figure 1) [99]. Among CD8^+^ T cells, intracellular TNF-α and IL-2 were significantly diminished in PD-1^+^TIGIT^+^ and PD-1^+^TIGIT^−^ cells as compared to PD-1^−^TIGIT^−^ cells, and the same trend was found for IFN-γ, indicating that TIGIT and PD-1 contribute to suppress the T-cell effector function. TIGIT is expressed by the majority of Tfh cells from the FL LN and by Tfh cells in tonsils from healthy donors (Figure 1) [99]. The TIGIT ligand either CD112 or CD155 was not expressed by tumor cells in all examined FL patients [99], but were expressed by FDCs in the FL microenvironment (Figure 1) [100]. TIGIT competes for ligand binding with the costimulatory receptor CD226. CD8^+^ effector memory T cells showed the highest expression of TIGIT but low expression of CD226. Contrary to CD8^+^ T cells, CD226 was often expressed in TIGIT^+^CD4^+^ T cells, including Tfh cells (Figure 1). This indicates that lower expression of CD226 on CD8^+^ T cells might play a role in TIGIT-mediated inhibition of CD8^+^ T cells in FL [99]. Upon ligation, TIGIT mobilizes the SH2-containing inositol 5-phosphatase 1 to alleviate the signals downstream of the adaptor protein SH2-domain containing leukocyte protein of 76 kDa, which results in ERK dephosphorylation and subsequent inhibition of IFN-γ production. Thus, TIGIT plays a role in dampening CD8^+^ T-cell antitumor response in FL. Moreover, Yang et al. showed that the higher levels of TIGIT on CD4^+^ T cells were attributed to the abundant expression of Treg and Tfh cells in FL. Furthermore, TIGIT preferentially expresses CD28^−^CD57^+^ or CD27^−^CD57^+^ CD8^+^ T cells, which suggests late-stage memory cells, defined as a population that exhibits an exhausted phenotype [101]. Finally, they demonstrated that increased numbers of TIGIT^+^ T cells related to inferior survival in FL patients [101]. Therefore, the immune checkpoint blockade targeting TIGIT enables a highly powerful T-cell antitumor response in several ways, by restoring the antitumor potential of T effector cells, dampening the Treg immunosuppressive effect, and diminishing the tumor-supporting effects of Tfh cells in FL.

Additionally, the lymphocyte-activation gene 3 (LAG-3) was found to be expressed on a subset of intratumoral T cells in FL, most of which were almost exclusively originated from the PD-1^+^ population (Figure 1) [102]. Intratumoral PD-1^+^LAG-3^+^ CD4^+^ or CD8^+^T cells exhibit reduced capability to secrete IFN-γ, IL-2, granzyme B, and perforin [89]. Moreover, the numbers of CD3^+^LAG-3^+^ or TIM-3^+^LAG-3^+^ cells were found to be correlated with poorer survival in FL patients [102].

Mass cytometry data further showed that intratumoral T cells lacking expression of CD27 and CD28 costimulatory receptor are enriched in FL and related to inferior OS in FL patients [103]. Moreover, CD70 is abundantly expressed in FL cells when compared to tonsil, and CD70^+^ lymphoma cells contribute to the expansion of CD27^−^CD28^−^ T cells This analysis demonstrated that both CD27 and CD28 are diminished in T cells cocultured with human lymphoma cells, which was reversed with an anti-CD70 antibody [103].

Recently, Pangault et al. proposed a tolerogenic niche for FL [104]. They compared the expression of *CD200* between MSCs, B cells, and Tfh cells between FL and tonsils, which revealed that *CD200* was significantly overexpressed in B cells and Tfh cells in FL LNs. In turn, CD200R was expressed on classical DC from FL LNs [104]. Therefore, CD200-CD200R engagement may confer an immunoregulatory signal, which results in the suppression of a T-cell-mediated immune response.

## 9. Conclusions

Considering the main players abovementioned CD4^+^ T-cell subpopulations supporting FL cells in the tumor microenvironment, Tfh and TFR cells could represent a therapeutic approach for the management of FL. Given the potential of the PI3K p110δ isoform inhibitor idelalisib simultaneously to inhibit proneoplastic cells in the FL microenvironment and to reduce the neoplastic B cells, Tfh cells and TFRs could reinforce the effects of the cytotoxic T cells. Hopefully, targeting EZH2 reprograms intratumoral Tregs; as for FL, namely, TFRs, which are more suppressive than Tregs in normal LNs, adoptive transfer of CAR-T cell therapy can be efficaciously executed even in the immunosuppressive tumor microenvironment. This combinatory strategy should be explored as a treatment options to tackle FL as armament in a straightforward direction instead of invigorating dysfunctional cytotoxic T cell therapies; otherwise, relapse will continue to persist among these FL patients.

## Figures and Tables

**Table 1:** Cell components in the microenvironment of follicular lymphoma.

Cells	Phenotype	Cytokines/Chemo-kine Production	Functions in FL	Reference
FRC (mice)	PDPN/gp38^+^ CD31^−^	IL-8 CXCL12,13 CCL2,7,19,21a (BAFF)	FL cell survival	[9]
FRC-like cells	-	IL-6,15, 33 CCL2, 5,11 CXCL10 (ICAM-1↑)	Tfh generation IL-4 production by Tfh (CXCR5^+^PD-1^dim^CD4^+^)	[24]
(cDCs)	CD11c^+^DCs	-	Assistance of Treg infiltration	[35]
FDCs	CCL21^+^CD23^+^↓	-	Diffuse growth pattern of FL	[36]
	CD21^+^	-	Shorten TTT, PFS, OS	[37]
	TGII^+^FRC/CD35^+^	(CMEs^lo^)	Reduction in FRCs/FDCs	[38]
	CD23^+^CD23^+^ERα^+^	-	Support of G1-2 FL microenvironment	[39]
	CD23^+^↓	-	More haphazard distribution of S-phase FL cells (i.e., G3 FL)	[40]
(HK cells)	(FDC-like cell derived from the human tonsil)	-	FL cell survival and proliferation	[51]
(HK cells)	-	CXCL12	Cancer stem cell-like activities	[49]
(HK cells)	-	IL-6, 8, CXCL1, 2, 5,12, CCL2	Angiogenesis, FL cell adhesion, migration, survival, and proliferation	[52]
Tfh	CD4^+^CXCR5^hi^CCR7^lo^ ICOS^+^PD-1^+^BCL6^+^	IL-2, 10 IFNγ	-	[59]
	CD4^+^CXCR5^hi^ ICOS^hi^PD-1^hi^ CD200^hi^ CD127/IL7-Rα^lo^	TNF-α IFNγ IL-4 (CD40L expression)	FL cell viability support FL cell viability support FL cell viability support and rescue from apoptosis	[60]
	CD4^+^PD-1^hi^ CXCR5^hi^BCL6^hi^	IL-4 IL-4 (CD40L expression)	pSTAT6 ↑ Treg-recruiting CCL17 and CCL22 production by FL cells	[61]
	CD4^+^CXCR5^hi^ PD-1^hi^ BCL6^+^	IL-4 (CD40L expression)	CXCL12↑i FL cell migration, adhesion to SCs, Syk and ERK phosphorylation	[56]
	CD4^+^PD-1^+^ICOS^+^	-	FL cell proliferation Increase in histological grade Immune synapse formation with Ki-67^+^ FL cells	[63]
	CD10^+^PAX5^−^ CD3^+^CD4^+^CXCR5^+^ PD-1^+^ICOS^+^ CXCL13^+^ HLA-DR^+^Ki-67^−^	IL-4^hi^IFNγ^lo ^ TNF-α^hi^ IL-21^hi^IL-17^lo^ (either CD10^+^ or CD10^−^)	B-cell activation and survival B-cell supportive lymphoid stromal network Treg-recruiting CCL17 and CCL22 production by FL cells	[65]
TFRs	CD4^+^CD25^+^ CXCR5^hi^ICOS^hi^ Blimp-1/PRDM1^+^	-	-	[60]
	CD4^+^CD25^+^ GITR^+^	-	Inhibition of FL LN-infiltrating T-cell cytokine production	[74]
	Bcl-6^+^ CXCR5^+^Foxp3^+^ BTLA^+^PD1^+^ICOS^+^ CD44^+^CTLA4^+^ GITR^+^	-	-	[10]
	CD4^+^Foxp3^+^Bcl-6^+^ Blimp-1/PRDM1^+^ CXCR5^hi^PD-1^hi^	-	-	[11]
	Foxp3^+^Ki-67^+^PD-1^+^ CXCR5^+^Bcl-6^+^	-	-	[12]
	CD4^+^CD25^+^GITR^+^ PD-1^dim^CXCR5^+^ Foxp3^+^	-	-	[75]
	CXCR5^+^ CD4^+^ PD-1^+^CD25^+^ BCL6^+^FoxP3^+^ CXCL13^+^ CTLA-4^+^IL-10^+^GITR^+^	CCL4 IL-16 CXCL13 (S1PR1↓SELL↓ CCR7↓)	Treg migration to the GC in response to a CXCL13 (CXCR5 ligand) gradient Chemotactic for CCR5-expressing Tregs Treg recruitment Treg retention in the GC More suppressive than normal LN Tregs	[76]
(TFR?)	CD25^hi^CD127^lo/-^ FoxP3^hi^ICOS^+^CXCR5^+^ CTLA-4^+^GITR^+^ CD39^+^PD-1^+^Ki-67^hi^ (activated Treg)	-	-	[77]

FRC, fibroblastic reticular cell; PDPN, podoplanin; gp, glycoprotein; CD, cluster of differentiation; IL, interleukin; CCL, CC chemokine ligand; CXCL, C-X-C motif chemokine; BAFF, B-cell-activating factor belonging to the tumor necrosis factor family; ICAM-1, intercellular adhesion molecule 1; ↑, upregulation; Tfh, T-follicular helper cells; CXCR5, C-X-C chemokine receptor type 5; PD-L1; programmed cell death ligand 1; cDCs, conventional DCs; TG, transglutaminase; ER, estrogen receptor; G, Grade; FDCs, follicular dendritic cells; TTT, time to transformation; PFS, progression-free survival; OS, overall survival; CMEs, collagen modifying enzymes; lo, low; ICOS, inducible T cell costimulator; PD-1, programmed cell death-1; BCL6, B-cell lymphoma 6; hi, high; pSTAT6, phosphorylated signal transducer and activator of transcription 6; CD40L, CD40 ligand; FL, follicular lymphoma; SCs, stromal cells; Syk, spleen tyrosine kinase; ERK, extracellular signal-regulated kinase; IFN-γ, interferon gamma; lo, low; CCL, CC chemokine ligand; Tregs, regulatory T cells; TFRs, T-follicular regulatory cells; Blimp-1, B lymphocyte-induced maturation protein-1; PRDM1, positive regulatory domain containing 1, with zinc finger domain; GITR, glucocorticoid-induced tumor necrosis factor-related protein; LN, lymph node; FoxP3, forkhead box protein 3; BTLA, B and T lymphocyte attenuator; CTLA-4^,^ cytotoxic T-lymphocyte-associated protein 4; S1PR1, sphingosine-1-phosphate receptor 1; ↓, downregulation; SELL, L-selectin; GC, germinal center; Th1,T helper1.

## Data Availability

Not applicable.

## Abbreviations

A2AR	adenosine 2A receptor
ABC	adenosine triphosphate-binding cassette
ABCC1	ABC subfamily C member 1
ABCG2	ABC sub-family G member 2
APRIL	a proliferation-inducing ligand
BAFF	B-cell-activating factor belonging to the tumor necrosis factor family
BCL2	B-cell lymphoma 2
BCL6	B-cell lymphoma 6
BCL-X_L_	B-cell lymphoma-extra-large
BM	bone marrow
B-NHL	B-cell non-Hodgkin lymphoma
BTK	Bruton’s tyrosine kinase
BTLA	B- and T-lymphocyte attenuator
CAFs	cancer-associated fibroblasts
CAR	chimeric antigen receptor
CCL	C-C motif chemokine ligand
CCR7	C-C chemokine receptor type 7
CD	cluster of differentiation
CD40L	CD40 ligand
CRCs	CXCL12-expressing reticular cells
CTLA-4	cytotoxic T-lymphocyte-associated protein 4
CXCL	C-X-C motif chemokine ligand
CXCR5	C-X-C chemokine receptor type 5
CTSS	cathepsin S
DCs	dendritic cells
FDCs	follicular dendritic cells
ERK	extracellular signal-regulated kinase
EZH2	epigenetic regulator zeste hololog 2
FL	follicular lymphoma
FoxP3	forkhead box protein 3
FRCs	fibroblastic reticular cells
GC	germinal center
GITR	glucocorticoid-induced tumor necrosis factor-related protein
HVEM	herpes virus entry mediator
ICAM-1	intercellular adhesion molecule 1
ICOS	inducible T-cell co-stimulator
ICOSL	ICOS ligand
IFN-γ	interferon-γ
IL	interleukin
ITIM	immunoreceptor tyrosine-based inhibition motif
KMT2D	lysin N-methyltransferase 2D
LAG-3	lymphocyte-activation gene 3
LFA-1	lymphocyte function-associated antigen-1
LN	lymph node
LT	lymphotoxin-α1β2
MedRC	medullary FRCs
MSCs	mesenchymal stem cells
MIP-1	macrophage inflammatory protein-1
MRCs	marginal reticular cells
OS	overall survival
PD-1	programmed cell death-1
PDPN	podoplanin
PFS	progression-free survival
PGE2	prostaglandin E2
p-STAT6	phospho-signal transducer and activator of transcription 6
R/R	relapsed or refractory
SH2	Src homology domain 2
SHP-1	SH2-containing protein tyrosine phosphatase-1
Syk	spleen tyrosine kinase
TAM	tumor-associated macrophages
Tfh	follicular helper T cells
tFL	transformed FL
TFRs	T-follicular regulatory cells
TG	transglutaminase
TIGIT	T-cell immunoglobulin and ITIM domain
TIM-3	T-cell immunoglobulin and mucin domain-containing protein 3
TNF	tumor necrosis factor
TNFRSF14	Tumor necrosis factor receptor superfamily 14
TRCs	T-cell zone reticular cells
Treg	regulatory T cell
TTT	time to transformation
VCAM-1	vascular cell adhesion molecule-1
